# First person – Luis E. Salazar Leon

**DOI:** 10.1242/dmm.050810

**Published:** 2024-06-12

## Abstract

First Person is a series of interviews with the first authors of a selection of papers published in Disease Models & Mechanisms, helping researchers promote themselves alongside their papers. Luis E. Salazar Leon is first author on ‘ Purkinje cell dysfunction causes disrupted sleep in ataxic mice’, published in DMM. Luis conducted the research described in this article while a PhD student in Roy V. Sillitoe's lab at Baylor College of Medicine, Houston, Texas, USA. After his PhD, he co-founded Sobek AI in Seattle, Washington, USA, and his current interests lie in using generative AI to accelerate and democratize scientific discovery.


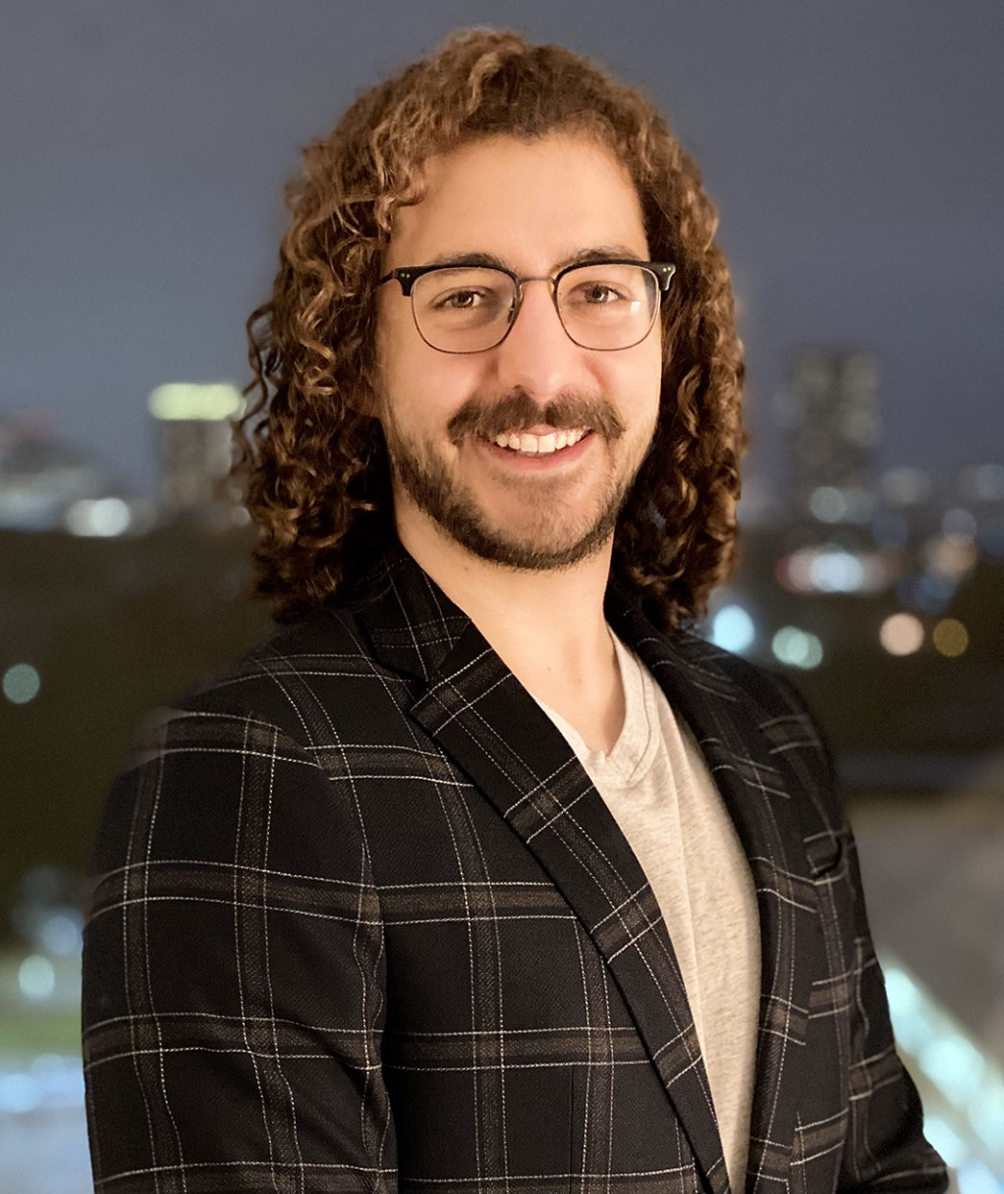


**Luis E. Salazar Leon**

**How would you explain the main findings of your paper to non-scientific family and friends?**

In our study, we investigated how the cerebellum, a part of the brain known for movement control, influences sleep. We focused on Purkinje cells, one of the primary cells in the cerebellum, using genetically modified mice in which these cells were silenced. Our findings showed that these mice had altered sleep patterns, including less rapid eye movement (REM) sleep (the lightest sleep stage), more non-REM sleep (the deepest sleep stage) and a delayed entry into REM sleep. This mirrors sleep problems in people with neurological motor disorders, suggesting the broader role of the cerebellum in sleep regulation. Our work highlights the complex interplay between different brain areas and their functions, emphasizing the significance of the cerebellum in sleep issues associated with neurological motor conditions. This could pave the way for new approaches to diagnosing and treating sleep disturbances in such disorders, challenging traditional perceptions of the limited role of the cerebellum in driving global brain states, such as sleep.

**What are the potential implications of these results for your field of research?**

Our study reveals a critical role for the cerebellum in regulating sleep, extending its known functions beyond movement control. We found that cerebellar disruptions could lead to sleep problems across a range of motor disorders, suggesting a common underlying mechanism. This discovery challenges traditional views and highlights the involvement of the cerebellum in the broader network dysfunctions associated with motor diseases. By pinpointing the impact of the cerebellum on sleep, our findings open new paths for treating sleep disturbances in neurological conditions, emphasizing the importance of addressing both motor and non-motor symptoms to improve patient care. This research underscores the interconnected nature of brain functions and the potential for novel therapeutic targets within the cerebellar circuitry, for instance, via deep brain stimulation.

**What are the main advantages and drawbacks of the experimental system you have used as it relates to the disease you are investigating?**

The genetically modified mice used in our experimental system benefit from precision in isolating the role of the cerebellum in sleep by specifically targeting Purkinje cells, the sole output neurons of the cerebellar cortex, providing a clear view of their role in sleep regulation. This specificity helps in understanding the direct impact of cerebellar dysfunction on sleep patterns. It also provides a controlled environment for clear data interpretation. However, the complexity of replicating the full spectrum of human conditions with genetic modification and the difficulty in translating findings from mice to human clinical applications are inherent drawbacks of using such a model.

**Figure:**
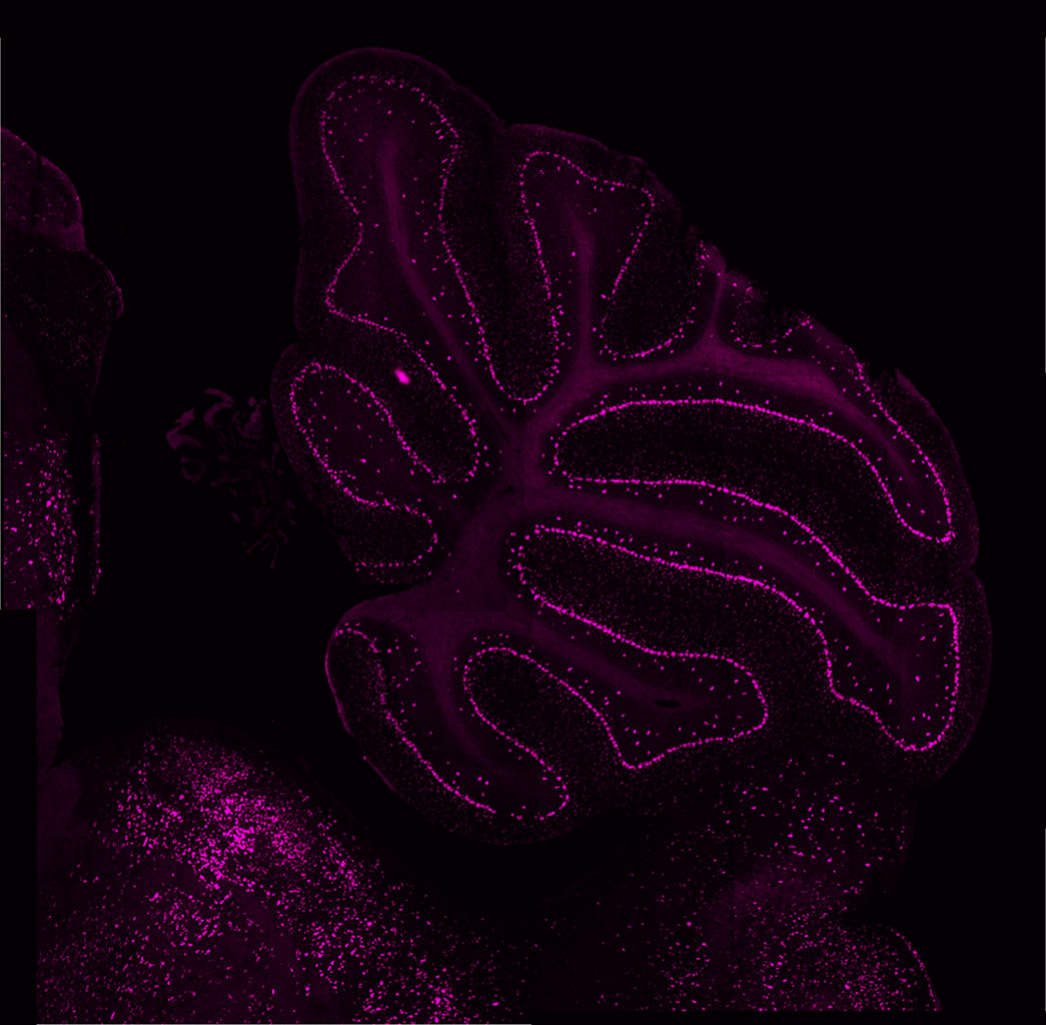
Immunostaining of the mouse cerebellum, labeling Purkinje cells in the cerebellar cortex.

**What has surprised you the most while conducting your research?**

The most striking revelation from our study was the widespread occurrence of sleep disturbances across a diverse range of cerebellar-related disorders, challenging the notion that these issues are confined to specific conditions. This insight shed light on the broader and more systemic influence of the cerebellum on neurological health than we had previously understood. Equally surprising was the realization of how overlooked the domain of sleep has been in cerebellar dysfunction research. Although perspectives are shifting, by recognizing sleep disturbances not merely as secondary symptoms but as integral aspects of neurological disorders, it was surprising to confront the knowledge gap in this area. This underscores a critical gap in our understanding and highlights the need for a paradigm shift in how we approach and study cerebellar impacts on sleep, and sleep dysfunction in general in the context of disease.

The capacity of AI to analyze complex, high-dimensional data and generate synthetic datasets that can mimic real-world biological phenomena will indeed revolutionize [research].

**What do you think is the most significant challenge impacting your research at this time and how will this be addressed over the next 10 years?**

I believe the most significant challenge impacting research currently is the availability of high-quality data, which is crucial for drawing reliable and actionable conclusions, especially in fields such as neuroscience where the complexity of the subject matter adds layers of difficulty in data collection and analysis. This challenge extends further to include aspects like data heterogeneity, privacy concerns and the need for large-scale, multidimensional datasets to reflect the intricate nature of fields like neuroscience.

Over the next 10 years, the advancement in artificial intelligence (AI) and synthetic datasets is expected to play a transformative role in addressing these challenges. The capacity of AI to analyze complex, high-dimensional data and generate synthetic datasets that can mimic real-world biological phenomena will indeed revolutionize this space.

However, this revolution will also require parallel advancements in computational methodologies, ethical guidelines and cross-disciplinary collaborations to ensure that synthetic data accurately represent the underlying biology and can be ethically used for research. Moreover, it is essential to consider the integration of advanced imaging technologies, genomics and patient-reported outcomes to enrich the datasets further. The development of federated learning models, which allow for the analysis of decentralized data without compromising privacy, could also be crucial in overcoming the data-sharing hurdles that often impede research progress.

**What changes do you think could improve the professional lives of scientists?**

To significantly improve the professional lives of scientists, we must confront and mitigate the substantial opportunity costs entailed in pursuing a PhD and a career in academia. The lengthy duration required to obtain a PhD, often exceeding 5 years, coupled with relatively low compensation during and after this period, presents a daunting challenge. This combination not only deters talented individuals from entering the field but also imposes a financial and emotional strain on those who commit to this path. The most immediate solution lies in straightforward enhancements to compensation, aligning the financial rewards of a scientific career more closely with its societal value and the level of expertise required. Naturally, this would also lower the barrier for those who would thrive in science but are hindered by the financial burden they would incur. Beyond this more obvious solution, another improvement lies in bridging the gap between academic science and the private sector, particularly startups. This cross-pollination offers mutual benefits, including the application of academic knowledge in real-world scenarios, which can enhance the commercial viability and societal impact of scientific research. Moreover, engaging with the private sector enables scientists to gain invaluable experience in project management, time management and entrepreneurial skills, fostering a more versatile and robust skill set that is applicable across the entire spectrum of science, whether in academia, government institutes, non-profit organizations or the private sector.

**What's next for you?**

After my PhD, I co-founded Sobek AI, a startup aiming to transform biopharma workflows with generative AI-driven tools, akin to a ‘GPS’ for biopharma teams. We are aiming to democratize global health innovation, translating collective knowledge and cutting-edge methodologies into actionable insights for those on the frontline of health advancements. Our platform empowers teams to navigate research challenges with confidence, advancing operational excellence and contributing to global health equity. This journey represents not just a step toward technological innovation, but a leap toward making a tangible difference in global health.
